# Effects of intravenous dextrose on preventing postoperative nausea and vomiting: A systematic review and meta-analysis with trial sequential analysis

**DOI:** 10.1371/journal.pone.0231958

**Published:** 2020-04-20

**Authors:** Chisaki Yokoyama, Takahiro Mihara, Shizuka Kashiwagi, Motokazu Koga, Takahisa Goto

**Affiliations:** 1 Department of Anesthesiology and Critical Care Medicine, Graduate School of Medicine, Yokohama City University, Yokohama, Japan; 2 Education and Training Department, Yokohama City University Hospital, YCU Center for Novel and Exploratory Clinical Trials, Yokohama, Japan; Geneva University Hospitals, SWITZERLAND

## Abstract

**Background:**

It is reported that postoperative nausea and vomiting, common general anesthesia complications, may be prevented by perioperative intravenous dextrose administration, but with controversial clinical effectiveness.

**Objective:**

To evaluate perioperative intravenous dextrose for preventing postoperative nausea and vomiting through a systematic review and meta-analysis of randomized controlled trials with trial sequential analysis.

**Data sources:**

MEDLINE, the Cochrane Central Register of Controlled Trials, Embase, Web of Science, clinicaltrials.gov, and the University Hospital Medical Information Network Clinical Trials Registry were searched from inception until 22 June 2019.

**Eligibility criteria:**

Trials investigating intravenous dextrose effects vs. placebos on postoperative nausea and vomiting in patients who underwent general anesthesia.

**Results:**

Eleven trials (1,250 patients) were included. All participants were ASA1-2. The nine trials included laparoscopic surgeries, and 92.2% of the participants were women. The timing of dextrose infusion was before, during, and after surgery in three, five, and three trials, respectively. Our results revealed intravenous dextrose administration significantly reduced postoperative nausea, but not vomiting, during early and late postoperative periods (risk ratio [95% confidence interval], early nausea: 0.76 [0.59–0.99], late nausea: 0.65 [0.48–0.89]; early vomiting: 1.00 [0.81–1.25], late vomiting: 0.96 [0.43–2.16]). Evidence quality was downgraded to low because the trial sequential analysis indicated more trials are needed for firm conclusions.

**Conclusions:**

Compared with placebos, perioperative intravenous dextrose administration may decrease postoperative nausea but not vomiting.

**Trial registration:**

University Hospital Medical Information Network Clinical Trials Registry (registration number: UMIN000030901).

## Introduction

Postoperative nausea and vomiting (PONV) are common complications associated with general anesthesia with an incidence of approximately 80% in a subset of high-risk patients. [1] PONV is a leading cause of aspiration pneumonia, dehydration, electrolyte imbalance, and patient dissatisfaction. Unresolved PONV may result in a prolonged hospital stay, unanticipated admissions, and prolonged nursing care. [2] Therefore, PONV prevention is important not only for improving patient safety, but also for reducing medical costs.

There are several pharmacological PONV prophylactic strategies, such as serotonin 5-HT_3_ receptor antagonists, dexamethasone, or droperidol administration. [1] However, these approaches implicate potential side effects and extra cost. For example, serotonin 5-HT_3_ receptor antagonists reduce PONV incidence, [3,4] but increase the incidence of arrhythmia, [5] headaches, [6] or dizziness. [7] Furthermore, serotonin 5-HT_3_ receptor antagonists are relatively expensive, thereby increasing treatment costs.

Intravenous dextrose administration may prevent PONV as it reduces insulin resistance and decreases gastric acid secretion, [8,9] which may contribute to PONV. Several studies have reported that perioperative dextrose administration decreases PONV, but its efficacy is controversial. [10–12] Therefore, we conducted a systematic review and meta-analysis to elucidate the effects of perioperative intravenous dextrose on PONV prevention.

## Methods

This study was a systematic review and meta-analysis with trial sequential analysis (TSA).

We followed the recommendations of the Preferred Reporting Items for Systematic Reviews and Meta-Analyses (PRISMA) statement and the Cochrane Handbook. [13,14] Our study protocol and analysis methods were prespecified and registered in the University Hospital Medical Information Network Clinical Trials Registry (registration number: UMIN000030901).

### Search strategy

The literature databases MEDLINE, Cochrane Register of Controlled Trials, Embase, and Web of Science were searched without language restrictions. Further, we endeavored to identify ongoing studies from clinicaltrials.gov and the University Hospital Medical Information Network Clinical Trials database. The reference lists of the retrieved full articles were also searched. The search strategy combining free text and Medical Subject Headings terms for PubMed is described in S1 File. The database search was conducted on June 22, 2019. All records were searched from database inception.

Two authors (CY and SK) independently scanned titles and abstracts of reports identified using the search strategies described above. If eligibility could not be determined from the title or abstract, the full paper was retrieved. Potentially relevant studies were retrieved and full-text versions were evaluated. Articles that met the inclusion criteria, described below, were assessed separately by the 2 authors, and any discrepancies were resolved through discussion.

### Inclusion and exclusion criteria

We searched for all randomized controlled trials (RCTs) that tested the effects of intravenous dextrose compared with a placebo on postoperative nausea (PON) and/or postoperative vomiting (POV). We excluded case reports, comments, reviews, and animal studies. We excluded studies that did not include fluid administration control groups, considering it is difficult to differentiate the effect of fluid administration and dextrose infusion in such trials. If a study included more than one control group, we only included the data of the control group with fluid administration. Eligibility was not restricted by surgery type, anesthetic technique, or patient age.

### Primary and secondary outcomes

The primary outcome of this meta-analysis was the incidence of PON/POV during the early postoperative period (defined below). The secondary outcomes were the incidence of PON/POV during the late postoperative period, the need for rescue antiemetics, hyperglycemia incidence, and postoperative blood glucose levels. The definitions of early and late postoperative periods are described below. If the primary outcomes were missing in the article, we contacted the corresponding authors for clarification. The pre-specified primary and secondary outcomes were modified. The modification details are described in the supplementary information (S1 File).

### Data collection

A data collection sheet was created and included data on the number of patients in study; patient age; patient sex; patient American Society of Anesthesiologists physical status classification; type of anesthesia; surgery type; dextrose dose; incidence of PON and POV during the early and late postoperative periods; number of rescue antiemetics used within 24 hours after surgery; side effects (blood glucose level, hunger, and thirst); and funding information. Data of the early and late periods of the first postoperative 24 hours were collected separately. When the first postoperative 24 hours were divided into 2 time periods (e.g., 0–6 and 6–24 hours), the first time period was defined as the early period and the second time period as the late period. When the first postoperative 24 hours were divided into more than 2 time periods, the data representing postoperative hours 1–2 and 24 hours were defined as the early and late periods, respectively. Values originally provided as percentages were converted into actual patient numbers for analysis. Two authors (CY and MK) extracted the data independently from the studies included, then cross-checked the data. Disagreements were resolved by discussion between the 2 authors.

### Assessment of risk of bias in individual studies

The risk of bias was assessed as described by the Cochrane Handbook for Systematic Reviews of Interventions. [14] The risk of bias was evaluated in sequence generation; allocation sequence concealment; blinding of patients, health care providers, data collectors, and outcome assessors; incomplete outcome data; selective outcome reporting; and other bias.

The risk of bias was classified as “low”, “high”, or “unclear”. Two authors (CY and MK) evaluated the risk of bias independently. The risk of bias summary was categorized as “low” for RCTs with a low risk of bias in all domains, “high” for RCTs with a high risk of bias in at least 1 domain, and “unclear” for RCTs that were neither “low” nor “high” in the risk of bias.

### Assessment of quality of evidence

The quality of evidence for the main outcomes was evaluated using the Grading of Recommendations Assessment, Development, and Evaluation (GRADE) approach with GRADEpro software. [15] Evidence quality was judged based on the presence or absence of the following variables: limitations in study design, inconsistency, indirectness, imprecision of the results, and publication bias. The quality of evidence for the main outcomes was graded as very low, low, moderate, or high.

### Statistical analysis

Continuous data were summarized using the mean difference (MD) with a 95% confidence interval (CI). Dichotomous data were summarized using a risk ratio (RR) with a 95% CI. If the 95% CI included a value of 0 or 1 for continuous or dichotomous data, respectively, the difference was not considered statistically significant. We used a random-effects model (DerSimonian and Laird methods [16]) to combine the results of the studies. When the number of studies was small (i.e., <10), we used the Hartung-Knapp-Sidik-Jonkman adjustment [17] for the random-effects meta-analysis. Heterogeneity was quantified with the I^2^ statistic; significant heterogeneity was considered to exist when the I^2^ statistic exceeded 50%. The cause of statistical heterogeneity was explored via a subgroup analysis according to the following predefined factors when the I^2^ statistic exceeded 50%: (1) presence or absence of prophylactic antiemetics, (2) dextrose dose (more or less than 250 ml of 5% dextrose), or (3) surgery type. Forest plots were used to graphically represent and evaluate the effects of treatment. Small-study effects, assessed using a funnel plot and an Egger’s regression asymmetry test, [18] were considered positive if *P* < 0.1 in the regression asymmetry test. Sensitivity analyses were performed for the incidence of PON/POV according to the risk of bias, in which RCTs with a high risk of bias or published in abstract form only were excluded. For statistically significant differences in outcomes, the number needed to treat (NNT) was calculated to estimate the overall clinical impact of the intervention.

For the incidence of PON/POV, TSA was performed to correct for random error and repetitive testing of accumulating and sparse data. [19–22] TSA monitoring boundaries and required information size (RIS) were quantified, and adjusted Cis were calculated. The RIS should be regarded as a target sample size for a meta-analysis. The risk of a type 1 error was maintained at 5% with 90% power. We used diversity (D^2^) [23] as an estimator of heterogeneity for the RIS calculation. The RR of 0.75 for PONV incidence (i.e., 25% relative risk reduction) was considered clinically meaningful. If the TSA-adjusted CI included a value of 1, the evidence quality was downgraded for the outcome due to the imprecision of the results.

Statistical analyses were performed using the R statistical software package, version 3.3.0 (R Foundation for Statistical Computing, Vienna, Austria). TSA was performed using TSA viewer version 0.9.5.9 β (www.ctu.dk/tsa).

## Results

**Fig 1:**
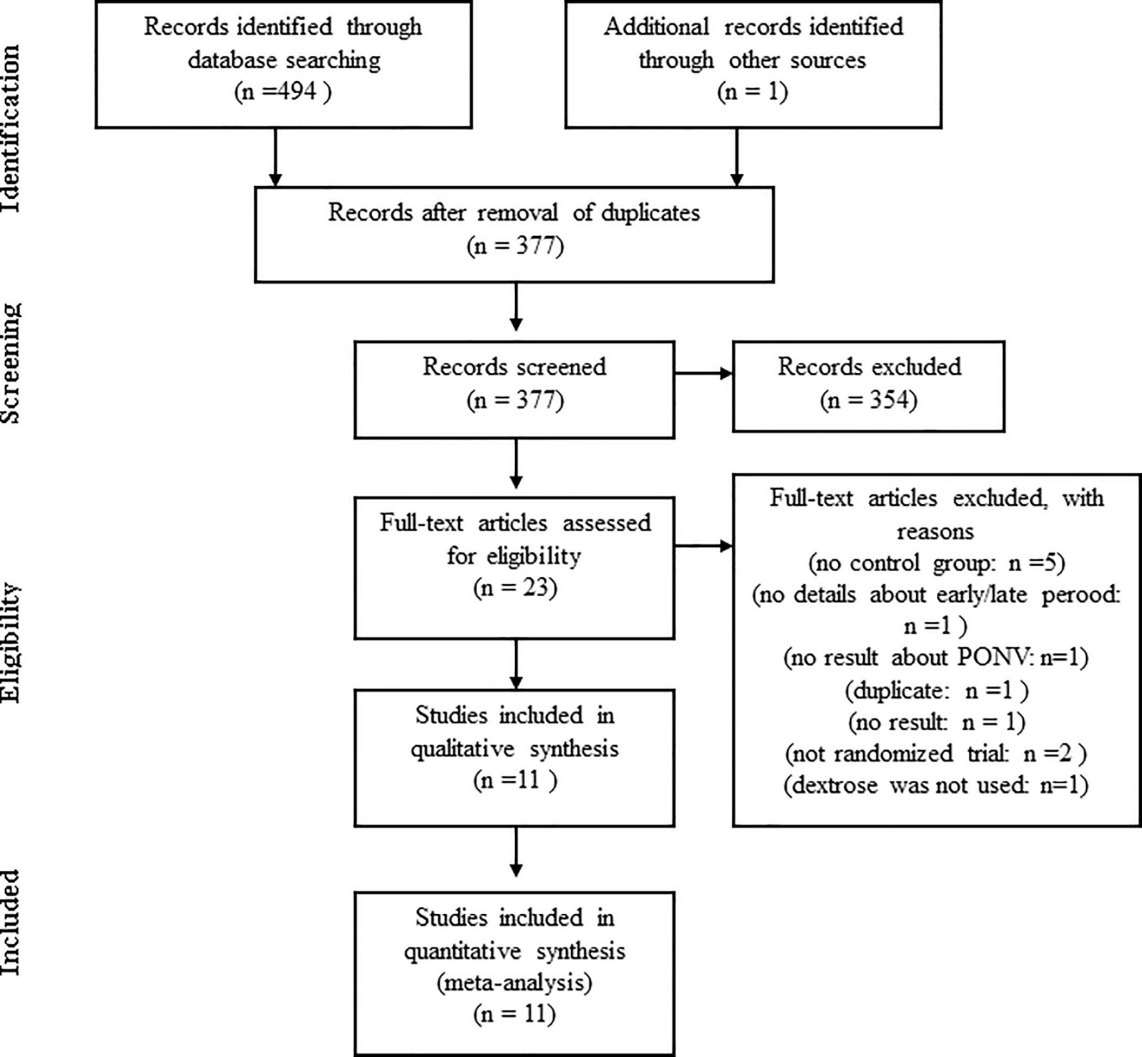
The PRISMA flow diagram for selection of studies.

A comprehensive search of the 6 databases produced 495 citations. The full texts of 23 articles were examined in detail. Twelve articles were excluded (S1 File) because they did not meet the inclusion criteria. Eleven articles with 1,250 patients were included in this meta-analysis (Fig 1). [10–12,24–31] Of the 11 articles, 10 were in English, and 1 was in Korean.

The features of the 11 RCTs are presented in Table 1. The participants were only women in 6 RCTs [11,12,24,27–29], and 92.2% of the participants in this meta-analysis were women. All participants included were ASA1–2. Table 1 presents the mean age of participants in each trial. The type of surgery in 9 RCTs was laparoscopic surgery. [10,12,24,25,27–31] In 8 RCTs, [12,24–29,31] the dextrose dose was > 12.5 g. In 3 RCTs, [10,12,31] prophylactic antiemetics were administered. One RCT [25] was an abstract-only publication. When the primary outcomes were missing in the article, we contacted the corresponding authors for clarification. However, we received no response.

**Table 1:** Characteristics of the included trials.

	Total number of patients	ASA-PS	Mean Age	Sex (M / F)	Surgery	Timing of administration	Dose of dextrose	Volume of dextrose	Length of infusion	Control fluid	Control Volume	Baseline antiemetics
Dabu-Bondoc 2013 [12]	62	1–2	37.5 (9.8)	0/62	Gynecologic laparoscopic and hysteroscopic procedures	After surgery	50 g	1000 mL	> 30 min	Ringer’s lactate solution	1000 mL	Ondansetron
Mishra 2017 [10]	100	1–2	39.6 (11.5)	28/72	Laparoscopic cholecystectomy	continuously during gallbladder removal	12.5 g	250 ml	From gall bladder removal to fluid finish	normal saline	250 mL	Dexamethasone
Patel 2013 [11]	162	1–2	45.5 (1.25)	0/162	Gynecologic, urologic, or breast surgery	Beginning with the start of surgical closure	12.5 g	250 ml	2 hours	Ringer’s lactate solution	250 mL	None
Conan McCaul 2003 [24]	120	1	32.3 (5.0)	0/120	Elective gynecological laparoscopy	At the induction of anesthesia	32 ± 5.8 g	1,148 ± 210 ml	20 min	CSL 1.5 mL/kg, no iv fluid	1,115 ± 363 mL	None
Firouzian 2017 [28]	150	1–2	41.4 (12.8)	0/150	Laparoscopic cholecystectomy	30 min before induction of anesthesia	25 g	500 ml	30 minutes	Ringer’s lactate solution	500 ml	None
Shin 2007 [26]	160	1–2	37.5 (14.7)	46/78	Not reported	Before anesthesia	0.1g/kg/hr, 1g/kg/hr	2 ml/kg, 20 ml/kg	30 minutes	Hartmann's solution	2 mL/kg, 20 mL/kg	Not reported
Rao 2017 [31]	115	1–2	43 (13)	unclear	Laparoscopic cholecystectomy	At the PACU	50 g	1000 mL	Within 30–40 minutes	lactated Ringer’s solution	1000 mL	Ondansetron
Cook 1990 [29]	75	1–2	31.4 (6.5)	0/75	Elective laparoscopic surgery	Before surgery	1 g/kg	20 mL/kg	45 minutes	no peri-operative fluid, 20 ml/kg compound sodium lactate solution	20 mL/kg	Not reported
Atashkhoei 2018 [27]	70	1	32.1 (1.0)	0/70	Diagnostic laparoscopy for infertility	5 minutes before the induction of anesthesia	500 mg/kg	10 mL/kg/hr	From 5 minutes before inducing anesthesia to the end of operation	Ringer’s solution with normal saline 0.9%	10 mL/kg/hr	Not reported
Jain 2016 [25]	150	1–2	Not reported	Not reported	Laparoscopic cholecystectomy	After surgery in the PACU	50 g	1000 mL	30–40 minutes	Ringer Lactate	1000 mL	Not reported
Pin On 2018 [30]	86	1–2	39.3 (10.7)	0/86	Gynecologic laparoscopy	During the anesthesia maintenance	unknown	2 mL/kg/hr	unknown	normal saline solution	2 mL/kg/hr	Not reported

ASA-PS, American Society of Anesthesiologists physical status; F, female; M, male; ACU, post-anesthesia care unit; SD, standard deviation

### Risk of bias

The risk of bias in the included trials is summarized in Table 2. No RCT was considered to have a low risk of bias, 3 RCTs were considered to have a high risk of bias, [25,26,28] and the rest had an unclear risk of bias. The details on each evaluation were reported in the S1 File.

**Table 2:** Risk of bias of the included trials.

	Sequence generation	Allocation concealment	Patients blinded	Health care providers blinded	Data collectors blinded	Outcome assessors blinded	Incomplete outcome data	Selective reporting	Other bias	Summary
Dabu-Bondoc 2013	Low	Low	Low	Low	Low	Low	Low	Unclear	Low	Unclear
Mishra 2017	Low	Low	Unclear	Low	Low	Low	Low	Low	Low	Unclear
Patel 2013	Low	Unclear	Low	Low	Low	Low	High	Low	Low	High
Conan McCaul 2003	Low	Unclear	Low	Unclear	Low	Low	Unclear	Unclear	Low	Unclear
Firouzian 2017	Low	Low	Low	Unclear	Low	Low	High	Low	Unclear	High
Shin 2007	Unclear	Unclear	Low	Low	Unclear	Unclear	High	Unclear	Low	High
Rao 2017	Unclear	Unclear	Unclear	Unclear	Unclear	Unclear	Low	Unclear	Unclear	Unclear
Cook 1990	Unclear	Unclear	Low	Low	Low	Low	Unclear	Unclear	Low	Unclear
Atashkhoei 2018	Low	Unclear	Low	Unclear	Low	Low	Unclear	Low	Low	Unclear
Jain 2016	Unclear	Unclear	Unclear	Unclear	Unclear	Unclear	Unclear	High	Unclear	High
Pin On 2018	Low	Unclear	Low	Unclear	Unclear	Unclear	Low	Unclear	Low	Unclear

### Early PON

**Fig 2:**
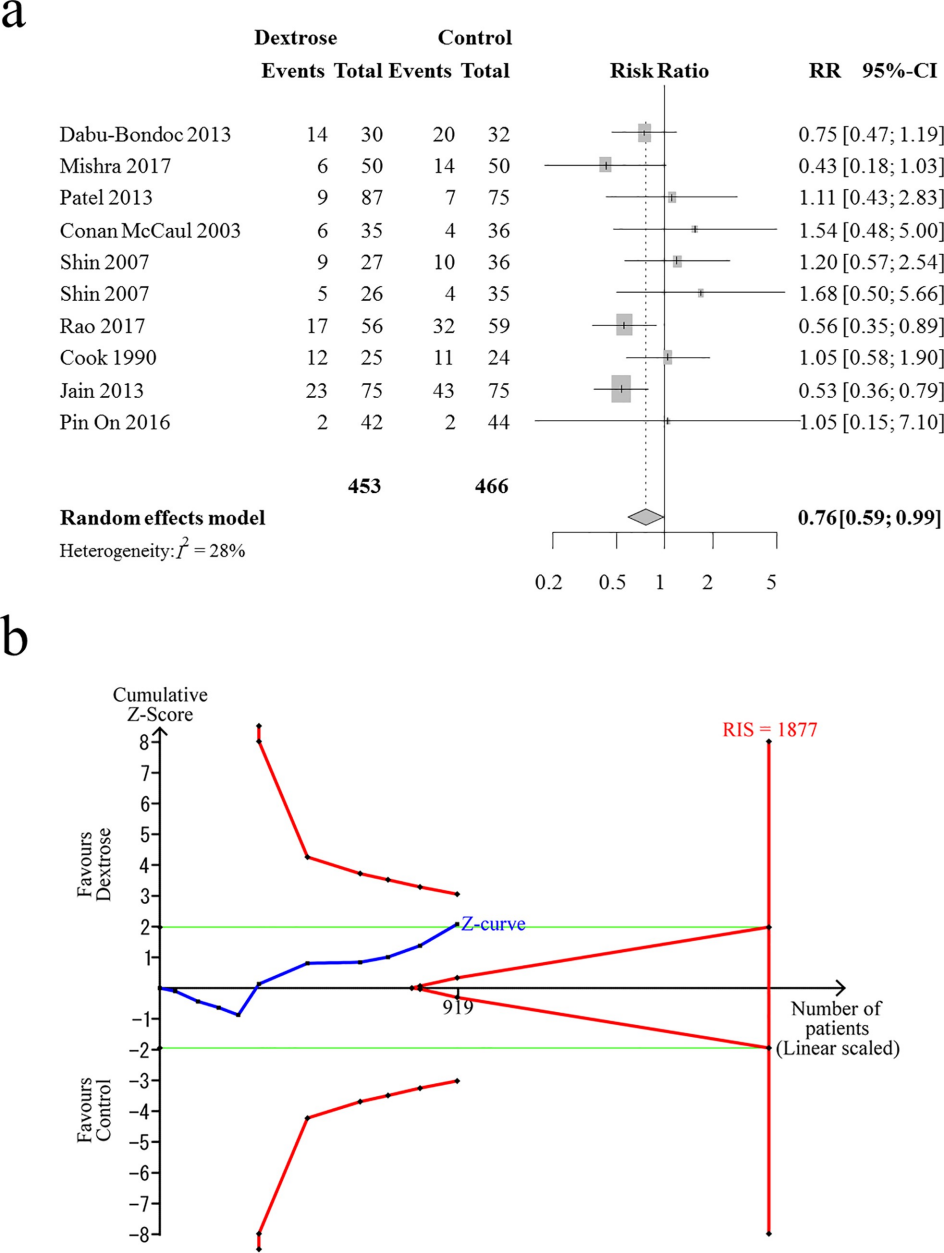
Intravenous dextrose effects on preventing postoperative nausea during the early postoperative period. A: Forest plot. B: Trial sequential analysis (TSA). The blue line is the cumulative Z-curve. The curved red lines are the TSA monitoring boundaries and the green lines are the conventional boundaries (i.e., *P* = 0.05) for benefit or harm. CI, confidence interval; RR, risk ratio; RIS, required information size.

Nine RCTs (10 comparisons) with 919 patients were analyzed for the effects of intravenous dextrose on the prevention of early PON. [10–12,24–26,29–31] Combined results showed that the incidence of early PON was significantly lower in the dextrose group than in the control group (RR [95% CI] = 0.76 [0.59 to 0.99], I^2^ = 28%, NNT [95% CI] = 15 [9 to 292], Fig 2A). A sensitivity analysis according to the risk of bias did not change the direction of dextrose effects on early PON prevention (RR [95% CI] = 0.84 [0.52 to 1.35], I^2^ = 9.4%). The Z-curve did not cross the TSA monitoring boundary (Fig 2B). The TSA-adjusted CI was 0.51–1.14 and the TSA revealed that the accrued information size (n = 919) was 49.0% of the RIS (n = 1877). A small-study effect was not detected using the regression test for funnel plot asymmetry (*P* = 0.11, S1A Fig). The evidence quality was downgraded to low due to the lack of precision and high risk of bias. The GRADE table with full details are presented in S1 Table.

### Early POV

**Fig 3:**
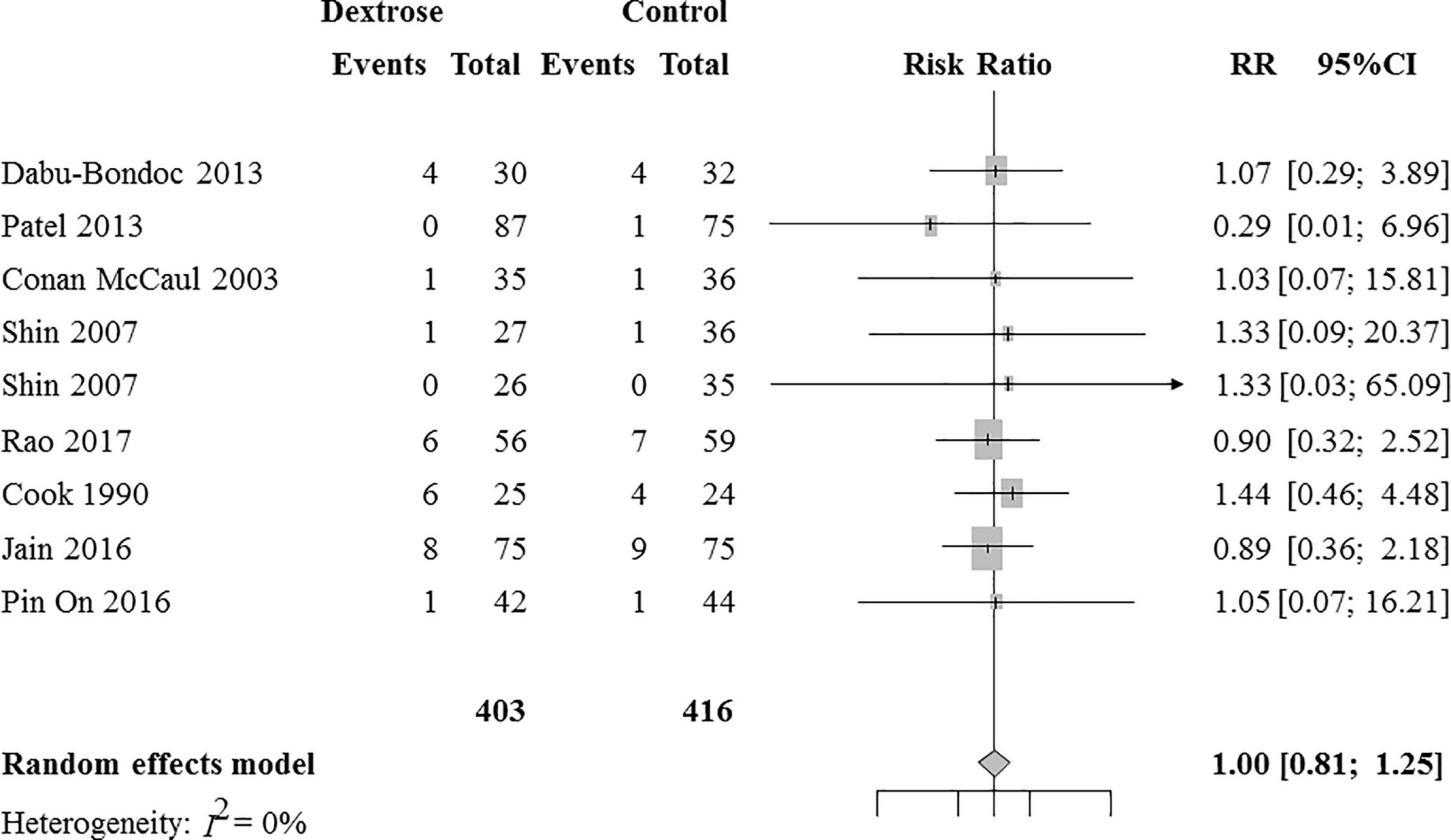
A forest plot for the effects of intravenous dextrose on preventing postoperative vomiting during the early postoperative period. CI, confidence interval; RR, risk ratio.

Eight RCTs with 819 patients were analyzed for the intravenous dextrose effects on early POV prevention. [11,12,24–26,29–31] Combined results showed that the incidence of early POV did not significantly differ between the dextrose and control groups (RR [95% CI] = 1.00 [0.81 to 1.25], I^2^ = 0%, Fig 3). A sensitivity analysis according to the risk of bias did not change the results (RR [95% CI] = 1.13 [0.56 to 2.28]).

The Z-curve did not cross the TSA monitoring boundary (S2 Fig); the TSA-adjusted CI was 0.14 to 7.49. TSA revealed that the accrued information size (n = 819) was only 9.8% of the RIS (n = 8321). A small-study effect was not detected with the regression test for funnel plot asymmetry (*P* = 0.92, S1B Fig). The quality of evidence was downgraded to low due to the lack of precision and the very small size of accrued information (S1 Table).

### Late PON

**Fig 4:**
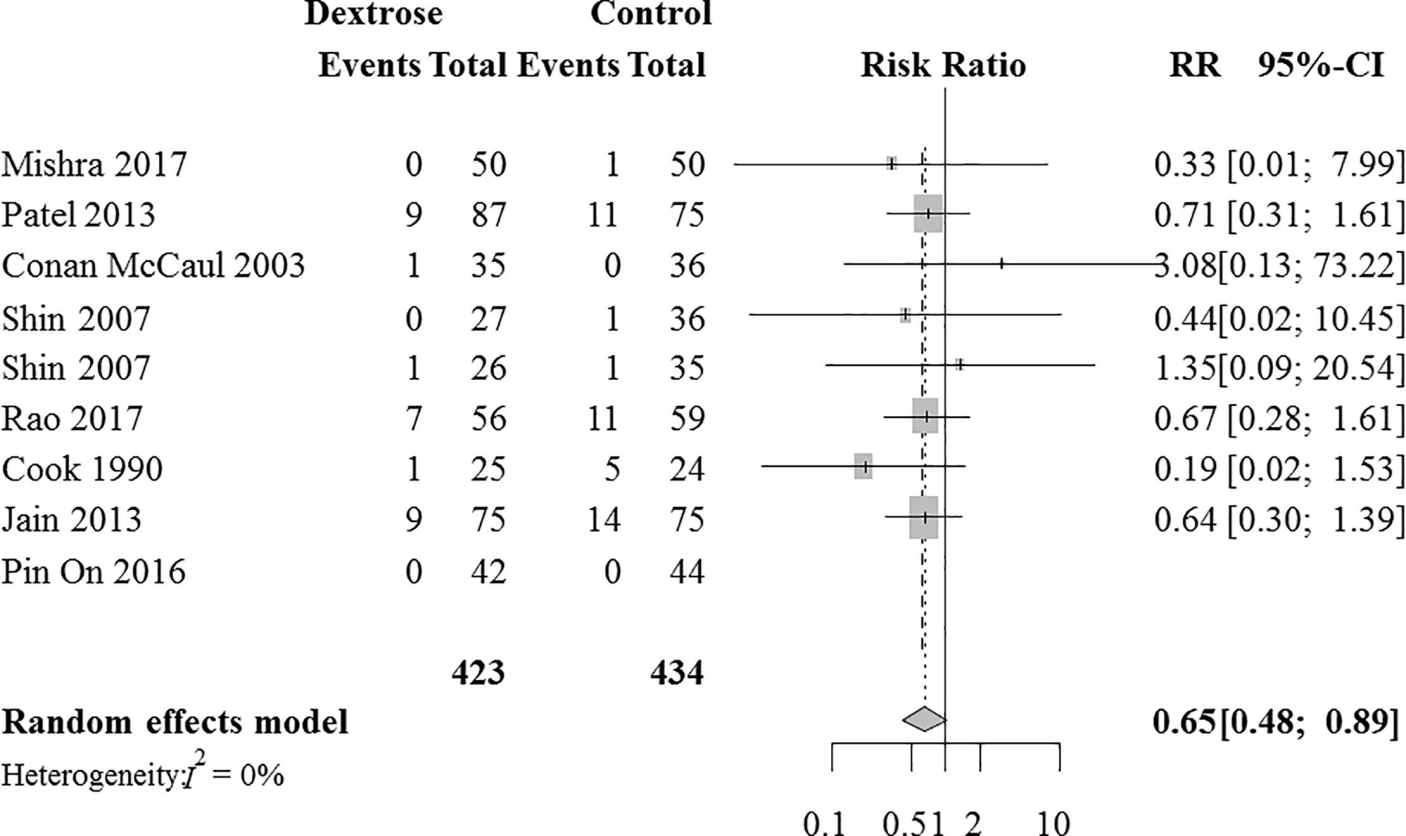
A forest plot for the effects of intravenous dextrose on preventing postoperative nausea during the late postoperative period. CI, confidence interval; RR, risk ratio.

Eight RCTs with 857 patients were analyzed for the intravenous dextrose effects on preventing late PON. [10,11,24–26,29–31] Combined results revealed that the incidence of late PON was significantly lower in the dextrose group than in the control group (RR [95% CI] = 0.65 [0.48 to 0.89], I^2^ = 0%, NNT [95% CI] = 31 [21 to 99], Fig 4). A sensitivity analysis according to the risk of bias did not change the direction of the dextrose effects on preventing late PON (RR [95% CI] = 0.62 [0.22 to 1.81]).

The Z-curve did not cross the TSA monitoring boundary (S3 Fig); the TSA-adjusted CI was 0.11 to 3.94. TSA showed that the accrued information size (n = 857) was only 12.5% of the RIS (n = 6838). A small-study effect was not detected using the regression test for funnel plot asymmetry (*P* = 0.76, S1C Fig). The quality of evidence was downgraded to low due to the lack of precision and the very small size of accrued information (S1 Table).

### Late POV

**Fig 5:**
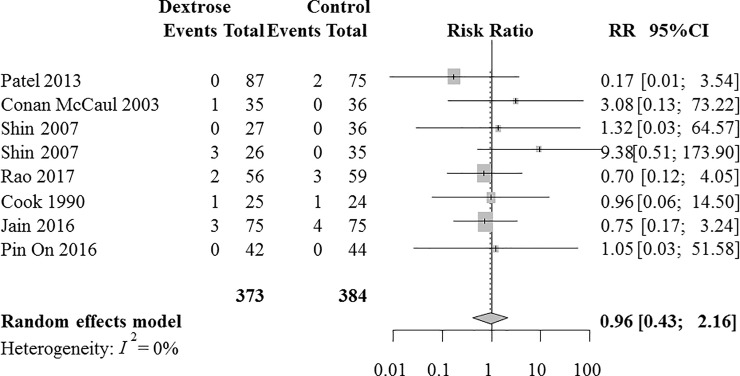
A forest plot of the effects of intravenous dextrose on preventing postoperative vomiting during the late postoperative period. CI, confidence interval; RR, risk ratio.

Seven RCTs with 757 patients were analyzed for the intravenous dextrose effects on preventing late POV. [11,24–26,29–31] Combined results revealed that the incidence of late POV did not differ between the dextrose and control groups (RR [95%] = 0.96 [0.43 to 2.16], I^2^ = 0%, Fig 5). A sensitivity analysis according to the risk of bias did not change the results (RR [95% CI] = 0.78 [0.02 to 24.5]).

A TSA boundary could not be calculated due to insufficient information (3.4% of the RIS). A small-study effect was not detected using the regression test for funnel plot asymmetry (*P* = 0.51, S1D Fig). The quality of evidence was downgraded to low due to lack of precision and the small size of accrued information (S1 Table).

### Rescue use

Eight RCTs with 750 patients were analyzed for the intravenous dextrose effects on the incidence of early rescue use. [10–12,24,27–29,31] Combined results revealed that the incidence of early rescue use did not differ between the dextrose and control groups, with high heterogeneity (RR [95% CI] = 0.70 [0.44 to 1.12], I^2^ = 70%, S4A Fig). A small-study effect was not detected with the regression test for funnel plot asymmetry (*P* = 0.14).

To explore the causes of heterogeneity, a prespecified subgroup analysis was conducted according to the dose of dextrose (more or less than 12.5 g). The incidence of early rescue use was not reduced in either high or low dose dextrose (RR [95% CI] = 0.65 [0.38 to 1.13], I^2^ = 40% and 0.71 [0.001 to 378], I^2^ = 91%, respectively, S4A Fig). The *P* value of the test for subgroup differences was 0.94. An analysis of the other prespecified subgroup was not conducted because the presence or absence of prophylactic antiemetics was not reported in 5 of 11 RCTs and only 1 RCT examined non-laparoscopic surgery.

Two RCTs with 186 patients were analyzed for the effects of intravenous dextrose on the incidence of late rescue use. [24,31] Combined results showed that the incidence of late rescue did not differ between the dextrose and control groups (RR [95% CI] = 0.90 [0.001 to 1315], I^2^ = 0%, S4B Fig). An asymmetry test could not be performed for the funnel plot because only 2 trials were included.

### Side effects

Six RCTs (7 comparisons) with 641 patients were analyzed for blood glucose levels after dextrose infusion. [11,26–29,31] Combined results revealed that intravenous dextrose infusions were associated with increased blood glucose levels compared to controls with high heterogeneity (MD [95% CI] = 65.9 [0.3 to 131.4] mg/dl, I^2^ = 100%, S5A Fig). To explore the causes of heterogeneity, a prespecified subgroup analysis was performed according to the dextrose dose (more or less than 12.5 g). Low-dose intravenous dextrose led to a significant but mild increase of blood glucose levels (MD [95% CI] = 32.5 [1.97 to 62.9] mg/dl, I^2^ = 0%, S5A Fig). High-dose intravenous dextrose did not significantly affect blood glucose levels (MD [95% CI] = 79.3 [-22.7 to 181.2] mg/dl, I^2^ = 100%, S5A Fig). The *P* value of the test for subgroup differences was 0.48.

Three RCTs (4 comparisons) with 288 patients were analyzed for hunger. [26,29,31] Combined results revealed that intravenous dextrose treatments significantly reduced the incidence of hunger compared to controls (RR [95% CI] = 0.73 [0.58 to 0.92], I^2^ = 0%, S5B Fig).

Three RCTs (4 comparisons) with 288 patients were analyzed for thirst. [26,29,31] Combined results revealed that the incidence of thirst did not differ between the treatment and control groups (RR [95% CI] = 1.28 [0.78 to 2.09], I^2^ = 72%, S5C Fig).

## Discussion

Our results indicate that intravenous dextrose administration reduces the incidence of PON, but not POV, during both the early and late postoperative periods (GRADE: low). However, the absolute reduction of the incidence of PON was small, and therefore, the NNT due to dextrose was high. Our results also suggest that intravenous dextrose increases blood glucose levels.

Intravenous dextrose administration is an approach used to prevent PON; we have shown that it reduced the incidence of early and late PON. The intravenous dextrose dose in 8 of the 11 included studies was ≥ 25 g, which is equivalent to 500 ml of 5% dextrose fluid. The administration timing was before or during surgery in 8 of the 11 included studies. However, the NNT for the effect of dextrose was high (NNT = 15 for early PON, NNT = 31 for late PON). Given that intravenous dextrose can cause hyperglycemia, it may be difficult to suggest routine administration, currently. Although the mechanisms of intravenous dextrose-mediated prevention of PON are not clear, we propose the following 3 hypotheses. First, high blood glucose levels due to intravenous dextrose may reduce gastric acid secretion, [32] consequently reducing gastric contractions and nausea. Firouzian et al. reported that nausea scores and blood glucose levels after post-anesthesia care unit arrival are negatively correlated. [28] Second, perioperative glucose administration may improve insulin resistance and may lead to a reduced incidence of PONV. [33–35] Third, dextrose and fluid administration may reduce pain and opioid consumption, [36] which lead to reducing PON. However, to elucidate the mechanism is out of the scope of the current review, and we cannot further examine the mechanisms with these methods. We focused on the effect of intravenous dextrose administration rather than oral intake. We considered intravenous administration to be a safe and efficient way to administer dextrose in surgical patients, especially those who cannot be orally fed. This is because all surgical patients have at least one intravenous route, all elective surgical patients are not allowed oral intake 2 hours before surgery for safety reasons, and some patients cannot orally intake medicines due to illnesses. However, since the perioperative administration of oral carbohydrates is known to reduce insulin resistance, oral glucose intake may reduce PONV. Furthermore, oral intake of dextrose is feasible in most patients undergoing laparoscopic surgery. Therefore, another systematic review for the effect of oral dextrose intake may be needed to elucidate its effect, although it is out of the scope of the current review.

Our results indicate that intravenous dextrose did not affect the incidence of POV, although it reduced the incidence of PON. One possible explanation for this discrepancy is the difference in pathogenesis between nausea and vomiting. Nausea is a subjective sensation evaluated by patients [37] and is regarded as a conscious cortical activity. [38] On the other hand, vomiting is regarded as a brainstem reflex. [38] Thus, any therapy for POV prevention should affect or modify brainstem activity. Based on our results, intravenous dextrose may not affect the brainstem though it likely affects cortical activity. Another possible explanation is the lack of statistical power in our meta-analysis of POV incidence. In our meta-analysis, the POV incidence in the control group was very low (6.7% and 2.6% in early and late periods, respectively). Consequently, the 95% CIs of the results were wide, precluding the ability to draw firm conclusions.

Our results show significant effects of intravenous dextrose on PON prevention, though this is inconsistent with a recent meta-analysis that reported perioperative intravenous dextrose had no significant effect on PONV reduction in the post-anesthesia care unit (RR [95% CI] = 0.91 [0.73 to 1.15], 7 RCTs with 661 patients) or within the first 24 h of surgery (RR [95% CI] = 0.76 [0.55 to 1.04]). [39] There are at least 2 possible reasons for this discrepancy. First, the recent meta-analysis assessed PON and POV together, [39] whereas we assessed PON and POV separately. As described above, the pathogenesis of nausea and vomiting differs. Notably, the recommendations for PONV research by Apfel et al. state that nausea and vomiting should be assessed separately. [38] Therefore, we assessed PON and POV separately, thereby reducing the heterogeneity of the outcomes. Second, the total sample size was larger in our study than in the recent meta-analysis. [39] We included 9 RCTs (10 comparisons) with 919 patients for analysis of early PON, whereas the recent meta-analysis included 7 RCTs with 661 patients. Thus, our larger sample size may have reduced beta errors.

The incident of early rescue use did not differ between dextrose and control groups. Subgroup analyses were conducted to elucidate the causes of heterogeneity observed in the overall analysis (I^2^ = 70%). However, the cause of heterogeneity was not detected because the 95% CIs were highly overlapped between the high and low dose dextrose and the *P* value of the test for subgroup difference (i.e., interaction *P* value) [40] was 0.94.

Hyperglycemia causes neutrophil dysfunction, decreases local immune responses at the surgical site, and increases the risk of surgical site infection (SSI). Particularly, several studies have reported that the risk of SSI increases with perioperative hyperglycemia. [41,42] In our meta-analysis, dextrose infusion increased blood glucose levels compared with control levels. Most RCTs showed a mild increase in blood glucose levels, but 2 RCTs using ≥ 50 g of dextrose reported blood glucose levels over 200 mg/dl after the intervention. [26,31] Although the incidence of SSI was not reported in all included RCTs, the results should be interpreted with caution because the majority of these studies excluded patients with diabetes mellitus. In the subgroup analysis, high-dose intravenous dextrose did not significantly increase blood glucose levels, whereas low-dose intravenous dextrose increased blood glucose levels significantly. Interpretation should be applied cautiously because the test for subgroup difference was not significant (interaction P-value = 0.48). Furthermore, the point estimate of the increase in blood glucose level was higher in the high-dose group.

Our meta-analysis has several limitations. First, the TSA indicated that the total sample size included in our meta-analyses was insufficient to draw firm conclusions. The Z-curve did not cross the TSA-monitoring boundaries in our primary outcome. Second, the incidence of PONV in the control group was low in our meta-analysis, especially in early POV and late POV, which may lead to a lack of statistical power. Thirdly, we modified the pre-specified primary outcome; this is a major limitation of this study. The pre-specified primary outcome was the incidence of PONV during postoperative 24 hours. Fourth, the trials included in our meta-analysis included only ASA1-2 patients and the majority of these studies excluded patients with diabetes mellitus. Further, patients with gastric paresis may not have been included in all studies. Therefore, our results can not apply to critically ill patients or patients with diabetes mellitus. Fifth, there was no RCT that had a low risk of bias. Therefore, we downgraded the evidence quality. We contacted authors to resolve unclear points regarding the risk of bias assessment, but we received no response. Sixth, heterogeneity was found in early rescue use, blood glucose level, and thirst. The cause of heterogeneity was not detected in our pre-specified subgroup analyses. Seventh, there were two RCTs [25,31] whose results in the Forrest plot were similar, and we could not firmly assess if the study population was overlapping between the two RCTs. We asked the authors if the same data were used in two articles, but received no response. We included two RCTs in our meta-analysis and confirmed that the sensitivity analysis, excluding the one RCT [25] with a high risk of bias, did not change the result.

In conclusion, perioperative intravenous dextrose administration may decrease the incidence of PON but not POV (GRADE: low). The incidence of early rescue antiemetics use did not differ between the dextrose and control groups.

## Supporting information

S1 Checklist Checklist of items to include when reporting a systematic review or meta-analysis. (DOC)

S1 File Supplemental digital content 1. (DOCX)

S1 Fig Funnel plots for early/late PON/POV. PON, postoperative nausea; POV, postoperative vomiting.
(TIF)

S2 Fig Trial sequential analysis for the effects of intravenous dextrose on preventing postoperative vomiting during the early postoperative period. The blue line is the cumulative Z-curve. The curved red lines are the trial sequential analysis monitoring boundaries and the green lines are the conventional boundaries (i.e., *P* = 0.05) for benefit or harm. RIS, required information size.
(TIF)

S3 Fig Trial sequential analysis for the effects of intravenous dextrose on preventing postoperative nausea during the late postoperative period. The blue line is the cumulative Z-curve. The curved red lines are the trial sequential analysis monitoring boundaries and the green lines are the conventional boundaries (i.e., *P* = 0.05) for benefit or harm. RIS, required information size.
(TIF)

S4 Fig Forest plots for the effects of intravenous dextrose on rescue use. a: The results for rescue use during early time periods. b: The results for rescue use during late time periods.
(TIF)

S5 Fig Forest plots for the side effects of intravenous dextrose. a: Results of blood glucose levels after administration. b: Results for hunger. c: Results for thirst.
(TIF)

S1 Table The GRADE table with full details. CI: Confidence interval; GRADE: Grading of Recommendations Assessment, Development, and Evaluation; RR: Risk ratio.
(DOCX)
