# Supplementary material for: Effects of intravenous dextrose on preventing postoperative nausea and vomiting: A systematic review and meta-analysis with trial sequential analysis
Source: PLoS One. 2020 Apr 20;15(4):e0231958. doi: 10.1371/journal.pone.0231958 (PMC7170240; doi:10.1371/journal.pone.0231958)
Supplement: S1 File — (DOCX) [file pone.0231958.s002.docx]

**Supplementary Information**

**PubMed Search Strategy**

("glucose"[MeSH Terms] OR "glucose"[All Fields] OR "dextrose"[All Fields]) AND (("postoperative nausea and vomiting"[MeSH Terms] OR ("postoperative"[All Fields] AND "nausea"[All Fields] AND "vomiting"[All Fields]) OR "postoperative nausea and vomiting"[All Fields] OR "ponv"[All Fields]) OR ((("postoperative period"[MeSH Terms] OR ("postoperative"[All Fields] AND "period"[All Fields]) OR "postoperative period"[All Fields] OR "postoperative"[All Fields]) OR ("surgery"[Subheading] OR "surgery"[All Fields] OR "surgical procedures, operative"[MeSH Terms] OR ("surgical"[All Fields] AND "procedures"[All Fields] AND "operative"[All Fields]) OR "operative surgical procedures"[All Fields] OR "surgery"[All Fields] OR "general surgery"[MeSH Terms] OR ("general"[All Fields] AND "surgery"[All Fields]) OR "general surgery"[All Fields]) OR ("surgical procedures, operative"[MeSH Terms] OR ("surgical"[All Fields] AND "procedures"[All Fields] AND "operative"[All Fields]) OR "operative surgical procedures"[All Fields] OR "surgeries"[All Fields]) OR ("anaesthesia"[All Fields] OR "anesthesia"[MeSH Terms] OR "anesthesia"[All Fields])) AND (("nausea"[MeSH Terms] OR "nausea"[All Fields]) OR retching[All Fields] OR ("vomiting"[MeSH Terms] OR "vomiting"[All Fields])))) AND (randomized controlled trial[pt] OR controlled clinical trial[pt] OR randomized[tiab] OR placebo[tiab] OR "drug therapy"[Subheading] OR randomly[tiab] OR trial[tiab] OR groups[tiab]) NOT ("animals"[MeSH Terms] NOT "humans"[MeSH Terms])

**The details on each evaluation for risk of bias domains**

| **Dabu-Bondoc 2013** |  |  |
| --- | --- | --- |
| Risk of Bias | Judgement | Reasons for the judgement |
| Sequence generation | Low | "Randomization was performed using the permuted block method" |
| Allocation consealment | Low | "Randomization was performed using the permuted block method with pharmacy-controlled allocation concealment." |
| Patients blinded | Low | "Intervention solution bags were placed in sequentially numbered, sealed, opaque black plastic bags to conceal group assignment from the subject, the anesthesia provider, and the PACU caregiver." |
| Health care providers blinded | Low | Intervention solution bags were placed in sequentially numbered, sealed, opaque black plastic bags to conceal group assignment from the subject, the anesthesia provider, and the PACU caregiver. |
| Data collectors blinded | Low | "To avoid interrater variability, an independent observer-investigator who was blinded to group assignment collected data in the PACU. " |
| outcome assessers blinded | Low | "To avoid interrater variability, an independent observer-investigator who was blinded to group assignment collected data in the PACU." |
| imcomplete outcome data | Low | "Sixty-four patients were enrolled in the study. Two patients were withdrawn after randomization because of extreme anxiety and abnormal blood glucose." |
| Selective reporting | Unclear | Insufficient information. |
| other bias | Low | Not detected |
| summary | Unclear | There is a unclear risk of bias in at least 1 domain and there is no high risk of bias. |

| **Mishra 2017** |  |  |
| --- | --- | --- |
|  | Judgement | Reasons for the judgement |
| Sequence generation | Low | " computer generated random number table." |
| Allocation consealment | Low | "Sealed opaque envelopes were opened in the OT and study fluid (normal saline or 5% dextrose) was decided according to the particular randomized group" |
| Patients blinded | Unclear | Insufficient information. |
| Health care providers blinded | Low | "For the purpose of blinding, the label of the study fluid (5% dextrose or normal saline) was removed by a senior anesthesiologist to prevent observer's bias. The person who was administering the study fluid was kept unaware about the type of study fluid." |
| Data collectors blinded | Low | "In the recovery room or in the ward, an independent observer who was not aware of the type of study fluid, noted the numbers of PONV episodes. " |
| outcome assessers blinded | Low | "In the recovery room or in the ward, an independent observer who was not aware of the type of study fluid, noted the numbers of PONV episodes. " |
| imcomplete outcome data | Low | No patient was withdrawn after randomization. |
| Selective reporting | Low | Almost all pre-registered outcomes were reported. |
| other bias | Low | Not detected |
| summary | Unclear | There is a unclear risk of bias in at least 1 domain and there is no high risk of bias. |

| **Patel P 2013** |  |  |
| --- | --- | --- |
|  | Judgement | Reasons for the judgement |
| Sequence generation | Low | "computerized randomization function" |
| Allocation consealment | Unclear | Insufficient information. |
| Patients blinded | Low | The anesthesia providers, surgeons, perioperative nurses, postanesthesia care unit (PACU) nurses, patients, and investigators were blinded to group assignment throughout. |
| Health care providers blinded | Low | The anesthesia providers, surgeons, perioperative nurses, postanesthesia care unit (PACU) nurses, patients, and investigators were blinded to group assignment throughout. |
| Data collectors blinded | Low | The anesthesia providers, surgeons, perioperative nurses, postanesthesia care unit (PACU) nurses, patients, and investigators were blinded to group assignment throughout. |
| outcome assessers blinded | Low | The anesthesia providers, surgeons, perioperative nurses, postanesthesia care unit (PACU) nurses, patients, and investigators were blinded to group assignment throughout. |
| imcomplete outcome data | High | Approximately 12％ of participants were excluded after randomization. |
| Selective reporting | Low | Almost all pre-registered outcomes were reported. |
| other bias | Low | Not detected |
| summary | High | There is a unclear risk of bias in at least 1 domain and there is no high risk of bias. |

| **ConanMcCaul 2003** |  |  |
| --- | --- | --- |
|  | Judgement | Reasons for the judgement |
| Sequence generation | Low | "Patients were randomized drawing unmarked envelopes containing assignment information." |
| Allocation consealment | Unclear | Insufficient information. |
| Patients blinded | Low | "Patients were randomized drawing unmarked envelopes containing assignment information and were blinded to their group allocation." |
| Health care providers blinded | Unclear | Insufficient information. |
| Data collectors blinded | Low | "Patients were assessed by a blinded interviewer using a standardized questionnaire in the postanesthe- sia care unit" |
| outcome assessers blinded | Low | "Patients were assessed by a blinded interviewer using a standardized questionnaire in the postanesthe- sia care unit" |
| imcomplete outcome data | Unclear | There was no data about the total number of included patients. |
| Selective reporting | Unclear | Insufficient information. |
| other bias | Low | Not detected |
| summary | Unclear | There is a unclear risk of bias in at least 1 domain and there is no high risk of bias. |

| **Firouzian 2017** |  |  |
| --- | --- | --- |
| Risk of Bias | Judgement | Reasons for the judgement |
| Sequence generation | Low | "computer-generated random numbers" |
| Allocation consealment | Low | A nurse anaesthetist who was blinded to the study groups allocated using a sealed-envelope technique. |
| Patients blinded | Low | Patients could not be aware of the allocation because placebo was used in the control group. |
| Health care providers blinded | Unclear | Insufficient information |
| Data collectors blinded | Low | "An anaesthesiology resident who was blinded to the study groups assessed the blood glucose level and PONV intensity for each patient." |
| outcome assessers blinded | Low | "An anaesthesiology resident who was blinded to the study groups assessed the blood glucose level and PONV intensity for each patient." |
| imcomplete outcome data | High | Approximately 20％ of randomised participants were excluded in the final analysis. |
| Selective reporting | Low | Almost all pre-registered outcomes were reported. |
| other bias | Unclear | Insufficient information |
| summary | High | There is a high risk of bias in at least 1 domain. |

| **Shin 2007** |  |  |
| --- | --- | --- |
|  | Judgement | Reasons for the judgement |
| Sequence generation | Unclear | Insufficient information. |
| Allocation consealment | Unclear | Insufficient information. |
| Patients blinded | Low | study fluid was covered by paper bag to blind patients and anestheologist. |
| Health care providers blinded | Low | study fluid was covered by paper bag to blind patients and anestheologist. |
| Data collectors blinded | Unclear | Insufficient information. |
| outcome assessers blinded | Unclear | Insufficient information. |
| imcomplete outcome data | High | After dextrose infusion, hyperglycemia was observed and experiment was discontinued. It decreased approximately 23% from the number of included patients. |
| Selective reporting | Unclear | Insufficient information. |
| other bias | Low | Not detected |
| summary | High | There is a high risk of bias in at least 1 domain. |

| **Rao 2017** |  |  |
| --- | --- | --- |
|  | Judgement | Reasons for the judgement |
| Sequence generation | Unclear | Insufficient information. |
| Allocation consealment | Unclear | Insufficient information. |
| Patients blinded | Unclear | Insufficient information. |
| Health care providers blinded | Unclear | Insufficient information. |
| Data collectors blinded | Unclear | Insufficient information. |
| outcome assessers blinded | Unclear | Insufficient information. |
| imcomplete outcome data | Low | Approximately 4％ of participants were excluded after randomization. |
| Selective reporting | Unclear | Insufficient information. |
| other bias | Unclear | Insufficient information. |
| summary | Unclear | There is a unclear risk of bias in at least 1 domain and there is no high risk of bias. |

| **Cook 1990** |  |  |
| --- | --- | --- |
|  | Judgement | Reasons for the judgement |
| Sequence generation | Unclear | Insufficient information. |
| Allocation consealment | Unclear | Insufficient information."Each patient was allocated at random to one of three groups." |
| Patients blinded | Low | "The intravenous fluid containers were covered by a large paper bag for concealment" |
| Health care providers blinded | Low | "The intravenous fluid containers were covered by a large paper bag for concealment" |
| Data collectors blinded | Low | "Assessments were made by one of the authors who was blinded to the treatment groups and did not participate in administration of the anaesthetic." |
| outcome assessers blinded | Low | "Assessments were made by one of the authors who was blinded to the treatment groups and did not participate in administration of the anaesthetic." |
| imcomplete outcome data | Unclear | Two participants were excluded after randomization but the reason was not reported. |
| Selective reporting | Unclear | Insufficient information. |
| other bias | Low | Not detected |
| summary | Unclear | There is a unclear risk of bias in at least 1 domain and there is no high risk of bias. |

| **Atashkhoei 2018** |  |  |
| --- | --- | --- |
|  | Judgement | Reasons for the judgement |
| Sequence generation | Low | "computer-generated randomization method" |
| Allocation consealment | Unclear | Insufficient information. |
| Patients blinded | Low | Patients could not be aware of the allocation because placebo was used in the control group. |
| Health care providers blinded | Unclear | Insufficient information. |
| Data collectors blinded | Low | Each result was recorded by the anesthesiologist who was blind to the study solutions. |
| outcome assessers blinded | Low | Anesthesiologist who was blind to the study solutions, was responsible for the post-operative management and recording of the study parameters. |
| imcomplete outcome data | Unclear | No information about exclusion. |
| Selective reporting | Low | Almost all pre-registered outcomes were reported. |
| other bias | Low | Not detected |
| summary | Unclear | There is a unclear risk of bias in at least 1 domain and there is no high risk of bias. |

| **Jain 2013** |  |  |
| --- | --- | --- |
|  | Judgement | Reasons for the judgement |
| Sequence generation | Unclear | Insufficient information. |
| Allocation consealment | Unclear | Insufficient information. |
| Patients blinded | Unclear | Insufficient information. |
| Health care providers blinded | Unclear | Insufficient information. |
| Data collectors blinded | Unclear | Insufficient information. |
| outcome assessers blinded | Unclear | Insufficient information. |
| imcomplete outcome data | Unclear | Insufficient information. |
| Selective reporting | High | The secondary outcomes were not reported. |
| other bias | Unclear | Insufficient information. |
| summary | High | There is a high risk of bias in at least 1 domain. |

| **Pin On 2018** |  |  |
| --- | --- | --- |
|  | Judgement | Reasons for the judgement |
| Sequence generation | Low | "computer-generated randomization process" |
| Allocation consealment | Unclear | Insufficient information. |
| Patients blinded | Low | Patients could not be aware of the allocation because placebo was used in the control group. |
| Health care providers blinded | Unclear | Insufficient information. |
| Data collectors blinded | Unclear | Insufficient information. |
| outcome assessers blinded | Unclear | Insufficient information. |
| imcomplete outcome data | Low | Approximately 7％ of participants were excluded after randomization. |
| Selective reporting | Unclear | Insufficient information. |
| other bias | Low | Not detected |
| summary | Unclear | There is a unclear risk of bias in at least 1 domain and there is no high risk of bias. |

**The list of major exclusions (12 studies)**

- Inadequate setting of control group or intervention group

Articles with oral CHS as intervention instead of iv dextrose^1,2^

Articles with intralipid as control^3^

Articles with fast as control^4^

Articles with injection of dextrose to P6 point as intervention instead of iv dextrose^5^

- Duplicate^6^
- Not Randomized trial^7,8^
- No result^9^
- Dextrose was not used^10^
- No result about PONV^11^
- No details about early/late period^12^

1. Libiszewski M, Drozda R, Smigielski J, Kuzdak K, Kolomecki K. Preparation of patients submitted to thyroidectomy with oral glucose solutions. *Pol Przegl Chir*. 2012; 84: 253-257.

2. Ozdemir F, Eti Z, Dincer P, Gogus FY, Bekiroglu N. The Effect of Preoperative Oral Carbohydrate Loading on Stress Response in Patients Undergoing Major or Minor Surgery. *Turkiye Klin Tip Bilim Derg*. 2011; 31: 1392-1400.

3. Ostman P, Faure E, Glosten B, Kemen M, Robert M, Bedwell S. Is the antiemetic effect of the emulsion formulation of propofol due to the lipid emulsion? *Anesth Analg*. 1990; 71: 536-540.

4. Cakar E, Yilmaz E, Cakar E, Baydur H. The Effect of Preoperative Oral Carbohydrate Solution Intake on Patient Comfort: A Randomized Controlled Study. *J perianesthesia Nurs Off J Am Soc PeriAnesthesia Nurses*. 2017; 32: 589-599.

5. Ravi M, Babu G, Somasekharam N, Dinesh M, Asha N, Hamsa J. Comparative efficacy of acupuncture at p6 point with 0.2ml 50% dextrose and inj ondansetron 50ug kg-1 iv for preventing postoperative nausea and vomiting. *J Anaesthesiol Clin Pharmacol*. 2010;26 CC-An:237-239.

6. Patel P, Nguyen M, Anderson DL, Rasmussen T, Brown J, Applegate RL. A closer look at the effect of dextrose on postoperative nausea and vomiting. *Anesth Analg*. 2011; 112.

7. Yang G-Z, Xue F-S, Li H-X, Liu Y-Y. Perioperative use of 5% dextrose to decrease postoperative nausea and vomiting. *J Clin Anesth*. 2017; 41: 63-64.

8. Castro-Alves LJ, Kendall MC. Dextrose for post-operative nausea and vomiting prophylaxis. *Indian J Anaesth*. 2018;62: 156.

9. Laws D. Morbidity associated with perioperative intravenous fluid in children undergoing tonsillectomy. http://apps.who.int/trialsearch/Trial2.aspx?TrialID=ISRCTN26015483. (accecced at January 20, 2018)

10. Cindea I, Balcan A, Gherghina V, et al. Effect of intraoperative nefopam on acute pain management after major abdominal surgery. *J Anaesth intensive care*. 2013; 20: 107-113.

11. Lee C, Kim H. The effects of preoperative dextrose loading on hyperalgesia induced by high-doses remifentanil in patients undergoing laparoscopy-assisted distal gastrectomy. *Eur J Anaesthesiol*. 2014; 31: 238-239.

12. Narayansa Irkal J, Reddy SV, Vardhan VH, Madhavi S. Role of dextrose on reducing postoperative nausea and vomiting following endoscopic middle ear surgery: a randomized, double-blind, controlled study. *Orig Res Artic Indian J Clin Anaesth*. 2016; 3: 352.

**All protocol violations**

1. The primary outcome

The primary outcome was modified. The pre-specified primary outcome was the incidence of PONV during postoperative 24 hours. The post-hoc primary outcomes were the incidence of PON/POV during the early postoperative period.

2. The secondary outcomes

The pre-specified secondary outcomes are the need for rescue antiemetics, incidence of hyperglycemia, postoperative blood glucose level. The post-hoc secondary outcomes were the incidence of PON/POV during the late postoperative period, the need for rescue antiemetics, hyperglycemia incidence, and postoperative blood glucose levels.

3. Date of search

We planned to search all databases at January 20, 2018. We initially searched all databases at January 20, 2018, and updated at June 22, 2019.

**Funding information of included trials.**

- Dabu-Bondoc 2013: Not reported
- Mishra 2017: Not reported
- Patel 2013: Departmental
- Conan McCaul 2003: Not reported
- Firouzian 2017: Grant
- Shin 2007: Not reported
- Rao 2017: None
- Cook 1990: Not reported
- Atashkhoei 2018: Not reported
- Jain 2016: Not reported
- Pin On 2018: Not reported
