# Supplementary figures and images for: Effects of intravenous dextrose on preventing postoperative nausea and vomiting: A systematic review and meta-analysis with trial sequential analysis
Source: PLoS One. 2020 Apr 20;15(4):e0231958. doi: 10.1371/journal.pone.0231958 (PMC7170240; doi:10.1371/journal.pone.0231958)

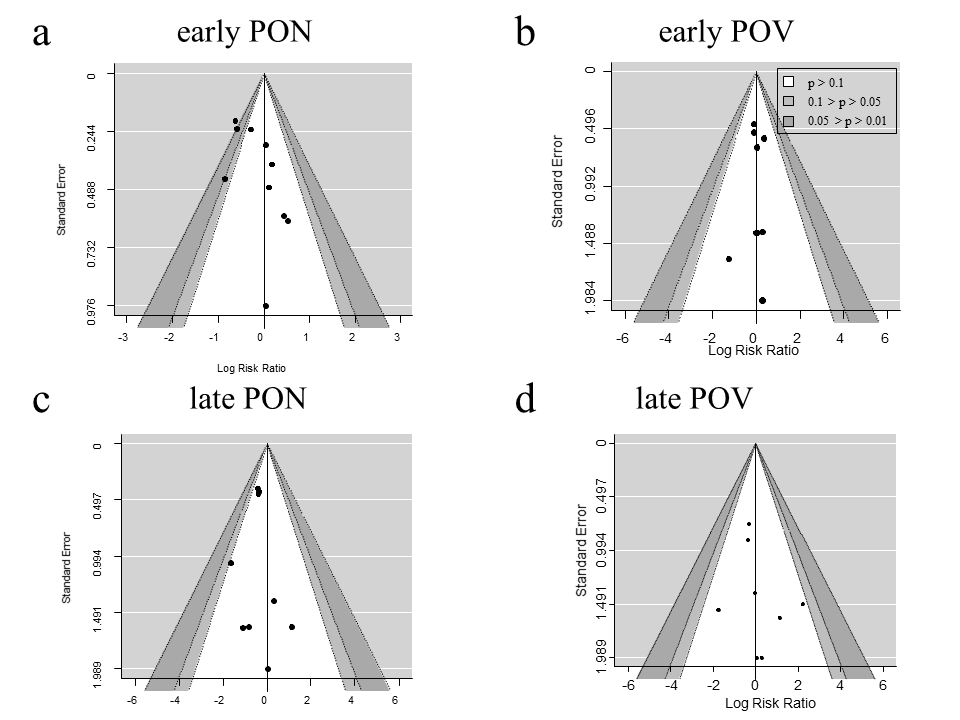

Supplement: S1 Fig — PON, postoperative nausea; POV, postoperative vomiting. (TIF) [file pone.0231958.s003.tif]

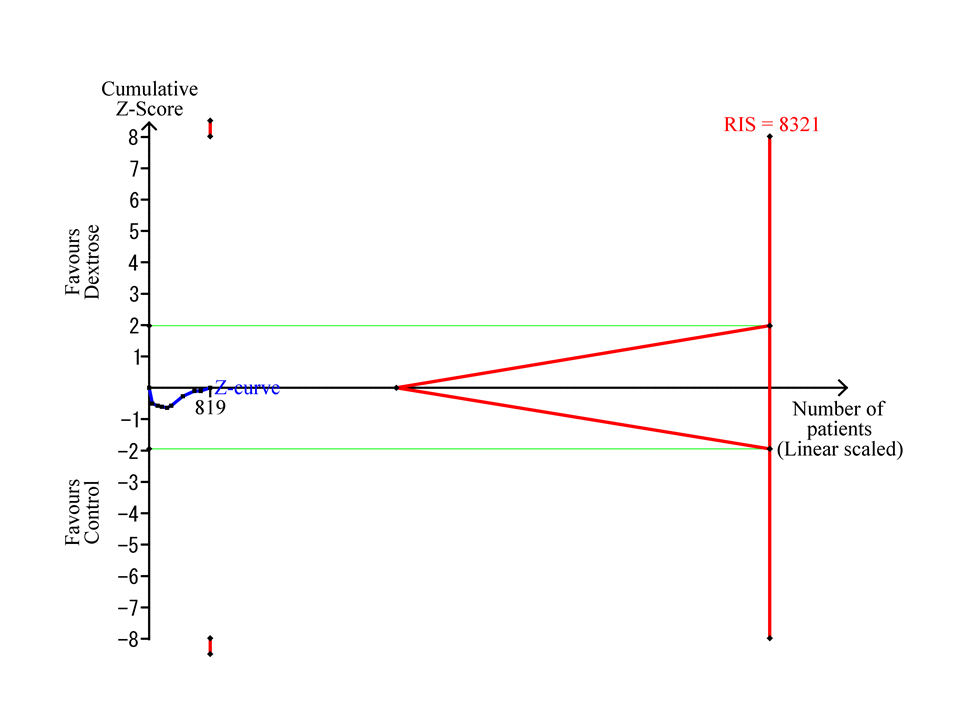

Supplement: S2 Fig — The blue line is the cumulative Z-curve. The curved red lines are the trial sequential analysis monitoring boundaries and the green lines are the conventional boundaries (i.e., P = 0.05) for benefit or harm. RIS, required information size. (TIF) [file pone.0231958.s004.tif]

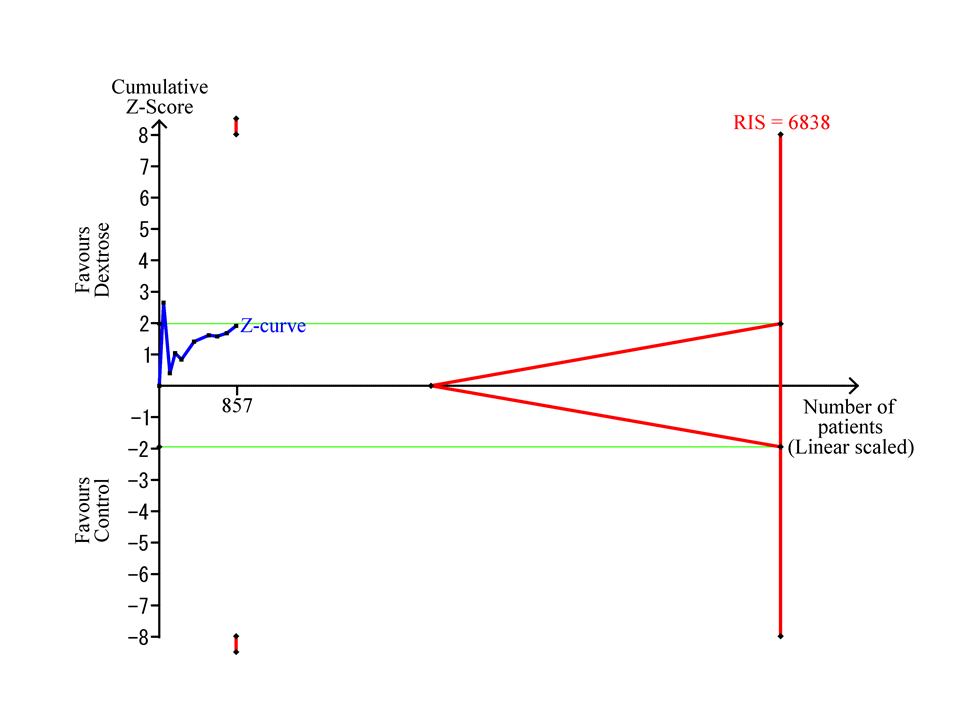

Supplement: S3 Fig — The blue line is the cumulative Z-curve. The curved red lines are the trial sequential analysis monitoring boundaries and the green lines are the conventional boundaries (i.e., P = 0.05) for benefit or harm. RIS, required information size. (TIF) [file pone.0231958.s005.tif]

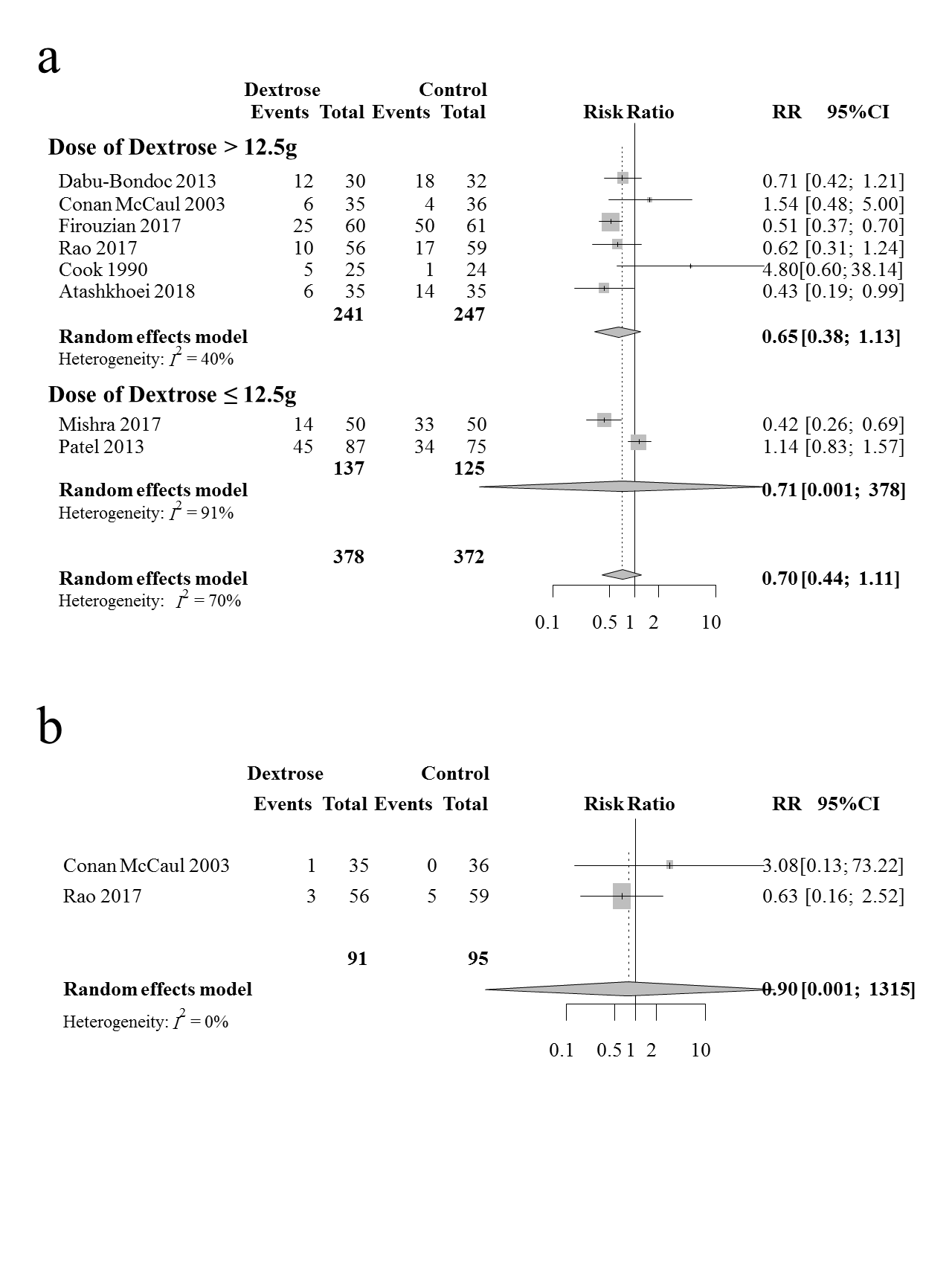

Supplement: S4 Fig — a: The results for rescue use during early time periods. b: The results for rescue use during late time periods. (TIF) [file pone.0231958.s006.tif]

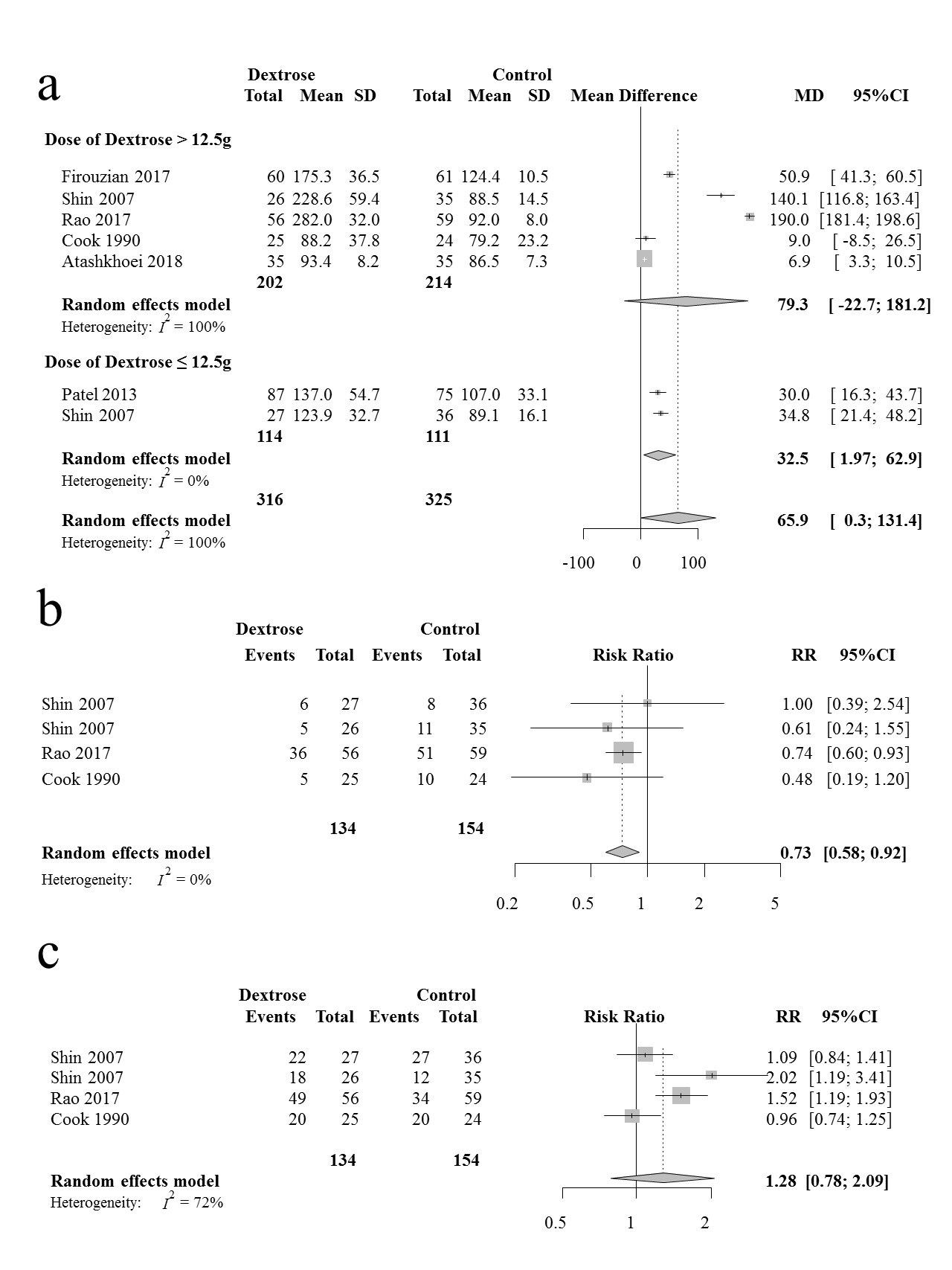

Supplement: S5 Fig — a: Results of blood glucose levels after administration. b: Results for hunger. c: Results for thirst. (TIF) [file pone.0231958.s007.tif]
