# Supplementary material for: Effects of intravenous dextrose on preventing postoperative nausea and vomiting: A systematic review and meta-analysis with trial sequential analysis
Source: PLoS One. 2020 Apr 20;15(4):e0231958. doi: 10.1371/journal.pone.0231958 (PMC7170240; doi:10.1371/journal.pone.0231958)
Supplement: S1 Table — CI: Confidence interval; GRADE: Grading of Recommendations Assessment, Development, and Evaluation; RR: Risk ratio. (DOCX) [file pone.0231958.s008.docx]

**Supplementary Table 1.** The GRADE table with full details.

| **Quality assessment** | | | | | | | **No of patients** | | **Effect** | | **Quality** | **Importance** |
| --- | --- | --- | --- | --- | --- | --- | --- | --- | --- | --- | --- | --- |
|  |  |  |  |  |  |  |  |  |  |  |  |  |
| **No of studies** | **Design** | **Risk of bias** | **Inconsistency** | **Indirectness** | **Imprecision** | **Other considerations** | **Intravenous dextrose administration** | **Control** | **Relative (95% CI)** | **Absolute** |  |  |
| **postoperative nausea during early time period** | | | | | | | | | | | | |
| 11 | randomised trials | no serious risk of bias | no serious inconsistency | no serious indirectness | serious^1^ | none | 103/453  (22.7%) | 147/466  (31.5%) | RR 0.76 (0.59 to 0.99) | 76 fewer per 1000 (from 3 fewer to 129 fewer) | ⊕⊕⊕O MODERATE | CRITICAL |
|  |  |  |  |  |  |  |  | 0% |  | - |  |  |
| **postoperative vomiting during ealy time period** | | | | | | | | | | | | |
| 9 | randomised trials | no serious risk of bias | no serious inconsistency | no serious indirectness | very serious^1,2^ | none | 27/403  (6.7%) | 28/416  (6.7%) | RR 1.00 (0.81 to 1.25) | 0 fewer per 1000 (from 13 fewer to 17 more) | ⊕⊕OO LOW | CRITICAL |
|  |  |  |  |  |  |  |  | 0% |  | - |  |  |
| **postoperative nausea during late time period** | | | | | | | | | | | | |
| 10 | randomised trials | no serious risk of bias | no serious inconsistency | no serious indirectness | very serious^1,3^ | none | 28/423  (6.6%) | 44/434  (10.1%) | RR 0.65 (0.44 to 0.89) | 35 fewer per 1000 (from 11 fewer to 57 fewer) | ⊕⊕OO LOW | IMPORTANT |
|  |  |  |  |  |  |  |  | 0% |  | - |  |  |
| **postoperative vomiting during late time period** | | | | | | | | | | | | |
| 8 | randomised trials | no serious risk of bias | no serious inconsistency | no serious indirectness | very serious^1,4^ | none | 10/373  (2.7%) | 10/384  (2.6%) | RR 0.96 (0.43 to 2.16) | 1 fewer per 1000 (from 15 fewer to 30 more) | ⊕⊕OO LOW | IMPORTANT |
|  |  |  |  |  |  |  |  | 0% |  | - |  |  |

^1^ The results of the trial sequential analysis showed that the Z-cumulative curve did not cross the Trial Sequential monitoring boundary.
^2^ The TSA showed that the accrued information size was only 9.8% of the targe sample size.
^3^ The TSA showed that the accrued information size was only 16.9% of the target sample size.
^4^ The TSA showed that the accrued information size was only 3.4% of the target sample size.

**CI:** Confidence interval; **GRADE:** Grading of Recommendations Assessment, Development, and Evaluation**; RR:** Risk ratio

GRADE Working Group grades of evidence
High quality: Further research is very unlikely to change the estimated effect.
Moderate quality: Further research is likely to have an important impact on the estimated effect and may change it.
Low quality: Further research is very likely to have an important impact on the estimated effect and is likely to change it.
Very low quality: There is considerable uncertainty regarding the estimate.
